# Effect of physical activity education on shoulder girdle pain and muscle strength in participants with fibromyalgia: a pilot experimental study

**DOI:** 10.3389/fpain.2024.1328796

**Published:** 2024-05-01

**Authors:** Bastien Couëpel, Catherine Daneau, Mathieu Tremblay, Thomas Javelot, Jacques Abboud, Isabelle Pagé, Martin Descarreaux

**Affiliations:** ^1^ Department of Human Kinetics, Université du Québec à Trois-Rivières, Trois-Rivières, QC, Canada; ^2^ Research Group on Neuromusculoskeletal Disorders (GRAN), Trois-Rivières, QC, Canada; ^3^ Department of Anatomy, Université du Québec à Trois-Rivières, Trois-Rivières, QC, Canada; ^4^ Department of Chiropractic, Université du Québec à Trois-Rivières, Trois-Rivières, QC, Canada; ^5^ Center for Interdisciplinary Research in Rehabilitation and Social Integration (Cirris), Centre Intégré Universitaire de Santé et de Services Sociaux de La Capitale-Nationale (CIUSSS-CN), Quebec, QC, Canada

**Keywords:** fibromyalgia, education, pain, muscle fatigue, shoulder girdle

## Abstract

**Background:**

In patients with fibromyalgia, exercise and education are recommended to decrease pain level and improve pain management. The latest scientific evidence recommends to focus interventions on the upper limb. The aim of this pilot study was to compare the immediate effect of physical activity education vs. a control group on pain and muscle capacity in fibromyalgia patients.

**Method:**

Fifty-six participants with fibromyalgia were randomized into an experimental group and a control group. The intervention consisted in watching a five-minute video that provided information about fibromyalgia, pain, kinesiophobia and physical activity. The control group watched a neutral five-minute video about beavers in Quebec. Following the video, participants performed a muscular fatigue task consisting of a repeated unilateral shoulder abduction task. At baseline and following the muscular fatigue task, maximal voluntary contraction (MVC) in shoulder abduction was assessed as well as pain level and pressure pain threshold (PPT) in the upper limb. Electromyographic activity was also assessed for upper trapezius and middle deltoid muscles. Two-way repeated measures analysis of variance was used to compare the MVC, PPT, and pain level before and after the muscular fatigue task between groups.

**Results:**

The experimental group showed a significantly lower increase in pain than the control group in the middle deltoid muscle (*p* = 0.002) when assessed by verbal pain rating scale. No significant interaction or main effect of Group and Time were observed for the pain level at the upper trapezius and elbow extensor muscles nor for any of the PPT measures. According to electromyographic data, the median frequency values indicate that neither group experienced muscle fatigue during the repeated contraction task.

**Conclusions:**

The preliminary results suggest that a short physical activity education video positively influenced middle deltoid pain following repeated abduction in participants with fibromyalgia. Electromyographic analysis showed no evidence of objective muscle fatigue, suggesting that there might be a partial disconnection between the perception of muscle fatigue and the physiological biomarkers associated with muscle fatigue.

## 1 Background

Fibromyalgia is a chronic pain condition affecting between 0.4% and 9.3% (average of 2.1%) of the general population and is among the first causes of chronic pain (1–3). This condition affects mainly women in their fifties, of low socioeconomic status, living in rural areas and those who suffer from obesity (3). There are still gray areas regarding the treatment of fibromyalgia, which result in an important socioeconomic challenge due to the annual cost of treatment, estimated between $1,250 and $8,504 annually per patient in Europe and between $4,085 and $5,264 annually per patient in Canada (4).

Fibromyalgia is characterized by chronic widespread pain and a complex polysymptomatology that involves fatigue, sleep disturbances and functional symptoms that cannot be explained by structural or pathological causes (1). Its definition has been expanded and updated by the American College of Rheumatology (ACR) over the past 30 years. In 1990, the ACR considered two main diagnostic factors: pain on the left and right sides of the body, above and below the waist (widespread pain), and the presence of at least 11 out of the 18 tender points characteristics of fibromyalgia (5). These criteria have been the subject of numerous discussions, particularly regarding the relevance and practicality of the tender points assessment. Following a revision of the ACR criteria in 2010 and 2011 (6, 7), the ACR proposed a final update in 2016, focusing on widespread pain, the severity of symptoms of fatigue (asthenia) and sleep disturbance (8). Fibromyalgia remains an enigmatic syndrome due to uncertainties regarding its diagnosis, its true origin, up to its management, which needs to be further explored (9). For this reason, it has been suggested to test the effectiveness of both medication and non-pharmacological treatments (10).

Our understanding of fibromyalgia's etiopathogeny is still incomplete (1) but there are considerable scientific evidence suggesting a central pain origin (11). Indeed, the phenomenon of central sensitization describes a dysfunction in the Central Nervous System (CNS), leading to an amplified response to mechanical stimulation through the enhancement of CNS signals. This, coupled with a reduced pain inhibition in patients with fibromyalgia (12–14), results in the development of allodynia and hyperalgesia. Lesnak and Sluka (13) have identified various factors characterizing the central sensitization phenomenon. The glutamate and substance *P* levels, an excitatory amino acid, appear to be higher in several brain areas of individuals with fibromyalgia, such as the posterior cingulate cortex. A high glutamate and substance *P* level could potentially explain alterations in pressure pain thresholds and the exaggeration of pain perception (1, 12). Additionally, dysfunction of the serotonin transporter, leading to a decrease in serotonin levels, is also linked to generalized hyperalgesia (12–14). These dysfunctions could be described as nociplastic alterations and represent a potential etiology of fibromyalgia (1).

Given its complexity, many questions remain regarding fibromyalgia pharmacological and non-pharmacological treatments (10). Pharmacological treatment, primarily consisting of nonsteroidal anti-inflammatory drugs (NSAIDs), antidepressants, antiepileptics, opioids, sedatives, and muscle relaxants, is used to manage the symptoms of fibromyalgia, albeit with mixed results and numerous side effects (15). Regarding non-pharmacological treatment, recent evidence suggest that physical activity and patient education have a positive impact on pain, and therefore improve the quality of life of patients with fibromyalgia (1, 16–19). Moreover, exercise therapy such as aerobic, strengthening and stretching exercises are highly recommended in the treatment of patients with fibromyalgia (20). High-intensity endurance training also seems to significantly improve the perception of pain and fatigue in this population (21).

Many patients complain of abnormal fatigue in tasks of daily living. Ickmans et al. (22) showed slower upper limb muscle recovery following muscular effort in participants with fibromyalgia compared to participants without fibromyalgia. Interestingly, persons suffering from fibromyalgia often report higher pain level in the shoulder and neck muscles than the arm muscles (23). Scientific evidence shows dysfunctions of the trapezius muscle such as mitochondrial disorders in type I fibers, hypotrophy of type II fibers, reduced capillarization and impaired microcirculation have also been observed in people with fibromyalgia (24, 25). For these reasons, researchers have recommended to focus future investigations on the upper limbs of patients with fibromyalgia (16).

According to Caneiro et al. (26) systematic review on belief about the body and pain, behavior change can only occur if the patient has the confidence to test some coping strategies on their own. Education, as part of a multicomponent approach for fibromyalgia, shows a level of evidence Ia and is recommended (strength of recommendation “weak”) by the European League Against Rheumatism (EULAR) (18). However, a systematic review showed significant effect on short-term pain reduction, together with a moderate clinical effect on the impact of fibromyalgia were observed after multiple sessions of education (27). For instance, after a 3 months follow-up, a health education program about pain physiology showed a significant positive effect on endogenous pain inhibition and health status of participants with fibromyalgia (28). *Fibro Friends* or *Amigos de Fibro* is a prime example of a multidisciplinary educational health promotion program tailored for individuals with fibromyalgia (29). This type of program has reached a high content validity index (CVI) score, allowing its utilization by primary health care professionals (30).

Considering the recommendations for exercise and education and given that muscular capacities of patients with fibromyalgia are inferior to healthy subjects, it was deemed interesting and innovative to measure the impact of physical activity education on pain and shoulder functional capacity in participants with fibromyalgia. The objective of this pilot study was to gather preliminary results regarding the immediate impact of physical activity education on pain and function in individuals with fibromyalgia. The purpose was also to identify unforeseen problems that could compromise the quality or flow of the study. Based on current knowledge (19, 26, 31), we hypothesized that physical activity education would positively influence pain and muscle fatigue in the shoulder girdle of participants with fibromyalgia. It was also hypothesized that physical activity education video would have an impact on the participants’ upper limb muscle strength. The results of this pilot study will be useful for the development of a full-scale randomized controlled study.

## 2 Methods

### 2.1 Study design

Based on recommendations of Johanson and Brooks (32), we aimed to recruit 24 to 36 participants per group. At last, fifty-six participants were recruited to assess the impact of a 5-minute physical activity education on pain and physical function in the shoulder muscles of participants diagnosed with fibromyalgia, all participants attended one experimental session.

### 2.2 Participants

Fifty-six participants (55 women/1 man; age: mean ± SD: 50.91 ± 10.04 years; height: 1.61 ± 0.06 m; weight: 78.50 ± 19.34 kg; BMI: 30.15 ± 6.52 kg/m^2^) were recruited between October 15 2021 and April 2022 for this study if they had been previously diagnosed with fibromyalgia according to the 2011 ACR. Participants were recruited through the *Association Fibromyalgie de la région du Québec* and the *Association de la Fibromyalgie-Mauricie Centre du Québec,* two local fibromyalgia associations*.* The testing sessions were conducted at the Department of Human Kinetics, Université du Québec à Trois-Rivières, Canada.

The inclusion criteria were the following: being between 18 and 70 years old and previuoulsy diagnosed with fibromyalgia by a physician (Widespread Pain Index (WPI) ≥7 and Symptom Severity Scale Score (SSS) ≥5 or WPI of 4–6 and SSS score ≥9) (6). The exclusion criteria were the following: participant reporting a history of any injury to the neck or upper limb region during the past six months, or unable to sustain a session of moderate to intense physical activity.

Written informed consent was obtained before the start of the study for each participant, in accordance with the ethics certificate granted by the ethics committee for research with humans from the Université du Québec à Trois-Rivières (CER-21-280.07-01).

### 2.3 Group allocation

**Figure 1:**
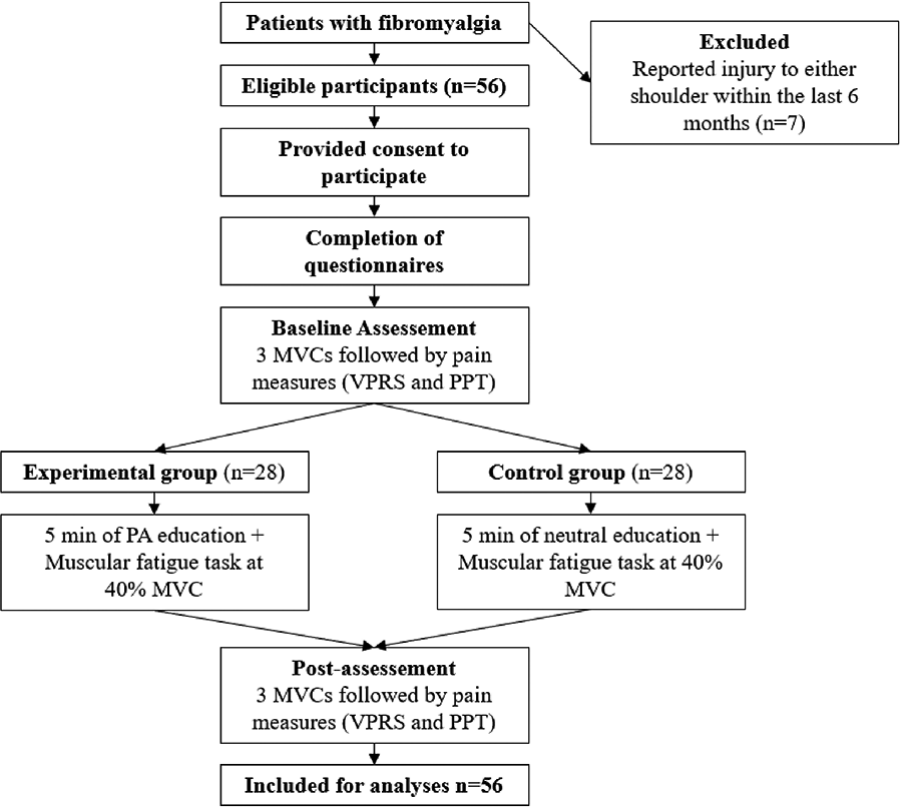
Experimental procedure. MVC, maximal voluntary contraction; PA, physical activity; VPRS, verbal pain rating scale; PPT, pressure pain threshold *Pain assessment was done at the end every series of muscular fatigue task.

Participants were randomly assigned to the experimental group (*n* = 28) or the control group (*n* = 28). The randomization process consisted in a computer-generated list of random numbers used to allocate participants to one of the two groups using a 1:1 ratio. An independent research assistant (M.D.) sequentially numbered envelopes containing intervention assignments according to the computer-generated randomization. Opaque and sealed envelopes were opened in front of the participants at the time of the experiment. Figure 1 illustrates the different stages of the study for each group.

### 2.4 Experimental and control interventions

The experimental group watched a custom designed 5-minute video intervention that provided general information about fibromyalgia, on the benefits of physical activity on pain and kinesiophobia, how to control pain during exercise. The video was divided into four parts: the first provided a definition of physical activity; the second discussed the beneficial effects of physical activity; the third addressed the theme of pain in physical activity, and the fourth explained to participants how to engage in physical activity considering their individual conditions. More precisely, it was explained that pain sensations are normal during physical activity, but that this pain should be controlled and sustainable, so that it does not persist after a physical activity session. The control group watched a 5-minute neutral video about beavers in Quebec for the control group, without any information regarding fibromyalgia or physical activity.

### 2.5 Repeated isometric muscle contraction task

Participants performed multiple series of submaximal arm abduction contractions immediately after watching the video, using a target force of 40% of previously evaluated MVC. One series included three 5-sec isometric contractions. The participant sat in the exact same position as during the MVC task. Visual feedback of the developed force was provided to the participants so they could reach the 40% MVC force target with a 5% margin of error. The task ended if the participant was no longer able to sustain the 40 ± 5% MVC force. At the end of the task, participants were asked to assess upper limb fatigue using a 11-point verbal rating scale at baseline and post-intervention (0 = no fatigue; 10 = maximal fatigue) (33).

### 2.6 Outcomes

#### 2.6.1 Baseline questionnaires

Five questionnaires were completed by all participants before the beginning of the experiment. The Tampa Scale of Kinesiophobia (TSK) was chosen to assess the level of kinesiophobia with a score out of 68. A score greater than or equal to 37 suggests the presence of kinesiophobia (34). The Disabilities of the Arm, Shoulder, and Hand (DASH) questionnaire was used to evaluate upper limb impairment with a score out of 100. This questionnaire focuses on the symptoms and the ability of participants to perform certain activities based on their conditions during the past week. A higher scores indicate a greater level of disability and severity with a minimal clinical important difference of 10.83–15 (35). For the Pain Catastrophizing Scale (PCS), assessing pain dramatization, participants were asked to indicate the extent to which pain-related thoughts and emotions are experienced with a score out of 52. A score exceeding 30 indicates a significant level of catastrophizing with clinical relevance (36). The Fibromyalgia Impact Questionnaire (FIQ) was used to measure the consequences of fibromyalgia on the health of participants with a score out of 100. A higher score indicate a greater impact of fibromyalgia (37). The Widespread Pain Index and Symptom Severity (WPI-SS) questionnaire was chosen to measure pain areas (score of 0–19) and symptom severity of fibromyalgia (score of 0–12) (7, 38).

#### 2.6.2 Maximal voluntary contraction (MVC)

The MVC test consisted in an isometric shoulder abduction performed in a sitting position, with the dominant arm in pronation (23, 39) and the elbow in full extension (40). The metallic grip used by the participant was attached to a load cell fixed to the ground (LSB350 model; Futek Advanced Sensor Technology Inc, Irvine, CA, USA). Deviation from the initial position was observed, and feedback regarding the correct arm positioning (45° of shoulder abduction) during the task was provided to participant to maintain the initial position. In this posture, the participants were instructed to pull on a metallic grip for 3–5 s using their maximum force. The three MVCs were separated by a minute of rest to prevent muscle fatigue. The highest value of force recorded over three trials was considered as the maximal force. During MVCs, verbal encouragement was provided to the participant (40). These MVCs were recorded before and after the muscular fatigue task.

#### 2.6.3 Surface electromyography (EMG)

Two bipolar surface electrodes (Model DE2.1, Delsys Inc., Boston, MA, USA) were used to record EMG activity during the baseline and post-intervention MVCs, and during the muscle fatigue task. Electrodes were positioned on the upper trapezius and the middle deltoid muscles on the dominant arm, following the muscle fibers orientation, at the level of the most prominent part of the muscle belly, according to SENIAM recommendations (41). Electrodes’ material was 99.9% Ag, and the inter-electrode distance was fixed at 10 mm. A reference electrode was positioned on same side acromion. Skin impedance on these three areas was reduced with: (1) shaving body hair, (2) gently abrading the skin with fine-grade sandpaper (Red Dot Trace Prep, 3 M; St. Paul, MN, USA) and (3) wiping the skin with alcohol swabs.

Bipolar EMG signals were amplified (DE2.1 model; Delsys, Inc., Boston, Massachusetts) by a factor of 1,000, sampled at 2,048 Hz and converted to digital form by a 12-bit A/D converter. Data were collected and processed with OT Bioelettronica (64-channel surface EMG amplifiers, SEA 64, LISiN-OT Bioelettronica; Torino, Italy; −3 dB bandwidths 10–500 Hz).

#### 2.6.4 Verbal pain rating scale (VPRS)

Participants were asked to rate their perceived pain on an 11-point verbal pain rating scale (VPRS) at baseline and post-intervention (0 = no pain; 10 = maximal pain) at the upper trapezius, middle deltoid, and elbow extensor muscles of their dominant arm (33). These two pain evaluations were used to assess the effect of the intervention following repeated upper-limb contractions.

#### 2.6.5 Pressure point thresholds (PPTs)

PPTs were assessed with a hand-held algometer (Model 01163, Manual Muscle Tester, Lafayette Instrument Company, USA) equipped with a 1 cm flat stirrup on the upper trapezius, middle deltoid, and elbow extensor muscles of their dominant arm (after MVCs, following verbal pain rating scale scores). PPT measurements were taken precisely at the midway point between the seventh cervical vertebra (C7) and the acromion for the upper trapezius (31), at the midpoint between the distal insertion of the deltoid and acromion for the middle deltoid (34) and at the bulge of the extensor carpi radialis muscle for the elbow extensor (35). Before the evaluation of PPTs, all participants were told the following sentences: “This is a test of your sensitivity to deep pain. Now I will press this pressure meter against your upper trapezius/middle deltoid/forearm and will gradually increase the pressure. Please say ‘Now’ as soon as the pressure starts to be painful. Remember that this is not a pain tolerance test, it is a pain threshold test.” (42). When the participant signaled the shift from pressure to pain, the algometer was promptly removed, and the force (measured in Kg/cm^2^) was recorded as the PPT value.

### 2.7 Data analysis

For PPT assessment, all measurements were done by the same researcher, twice at each muscle, and in the same order (upper trapezius, middle deltoid, and elbow extensor muscles). The average of the two measurements was used for the analyzes.

All EMG data were digitally band-pass filtered in the frequency bandwidth 20–450 Hz (2nd order Butterworth filter). Sixty hertz power line interference and its harmonics were reduced using notch filters (120, 180, 240, 300, 360, 420 Hz). To analyse muscular activity during MVC, root mean square (RMS) values were calculated using 500 milliseconds windows for each electrode, on the entire signal during each trial of MVCs. To identify the presence of muscle fatigue, RMS values were obtained before and after the repeated isometric contraction task, during MVCs. In addition, the median frequency (MDF) was calculated for the first and last 5 s of contraction during the muscular fatigue task for each electrode (43).

### 2.8 Statistical analysis

Normality of data set was verified by visual inspection and Shapiro-Wilk test. Participants’ characteristics (demographic and questionnaires) were compared using *t*-tests for independent measures or Mann-Whitney *U*-test. The Fisher's exact test was conducted to determine if the proportions of men and women as well as usual pharmacological treatments among the two groups were different. For non-normal distributed variables, the median and interquartile (Q1-Q3) were used. Two-way repeated measures analysis of variances (ANOVAs) were performed to evaluate the presence of a main effect of Group (experimental and control), Time (baseline and post-intervention) and Group*Time interaction for verbal pain rating scale, PPT scores and MVCs force. Post-hoc Bonferroni test for multiple comparisons was performed for significant differences.

Additionally, two-way repeated measures analysis of variances (ANOVAs) were performed to assess the main effect of Group (experimental and control), Time (at the beginning and at the end of the repeated isometric muscle contractions) and the Group*Time interaction for MDF and fatigue scores during the repeated isometric muscle contraction task.

The Mann Whitney *U*-test was performed on RMS values to compare baseline and post-intervention muscular fatigue during MVCs for each group. All analyses were conducted using Statistica (Tibco Software Inc. 2018. Tibco Statistica, Version 13.5.0.17, Palo Alto, California) and IBM SPSS Statistics version 28.0.0. Significance was set at *p* ≤ 0.05.

## 3 Results

### 3.1 Participant characteristics

Table 1 presents participants’ characteristics and indicates that no significant difference was found between groups. Every participant included in the study completed the protocol for the control and experimental group. The proportions of medication categories used by participants in both groups are presented in Table 1. According to the Fisher's exact test, there is no difference in proportions between the two groups for all medication categories.

**Table 1:** Participants’ baseline characteristics.

	Total (*n* = 56)	Experimental group (*n* = 28)	Control group (*n* = 28)	*P*-value
	Mean (± SD)	Mean (± SD)	Mean (± SD)	
Sex (F/H)	55/1	28/0	27/1	1^a^
Age (years)	50.91 (±10.04)	49.04 (±10.66)	52.79 (±9.18)	0.164
Weight (kg)	78.50 (±19.34)	80.90 (±20.66)	76.10 (±17.98)	0.358
Height (m)	1.61 (±0.06)	1.61 (±0.07)	1.62 (±0.06)	0.549
BMI (kg/m^2^)	30.15 (±6.52)	31.24 (±6.90)	29.07 (±6.04)	0.217
Diagnostic (years)	9.56 (±7.87)	7.81 (±6.29)	11.25 (±8.94)	0.106
TSK (/68)	44.77 (±6.27)	44.61 (±6.41)	44.93 (±6.15)	0.849
DASH (/100)	35.01 (±14.24)	37.97 (±14.48)	32.04 (±13.60)	0.120
PCS (/52)	24.84 (±10.79)	24.68 (±10.82)	25.00 (±10.96)	0.913
WPI (/19)	10.46 (±3.91)	10.71 (±4.25)	10.21 (±3.60)	0.637
SS (/12)	9 (8–10)^b^	9 (8–10)^b^	9.5 (8–10)^b^	0.818^c^
FIQ (/100)	53.24 (±13.04)	55.91 (±11.64)	50.56 (±13.99)	0.125
Antidepressant	30/56	17/28	13/28	0.422^a^
Anticonvulsant	14/56	8/28	6/28	0.768^a^
Analgesics	22/56	11/28	11/28	1^a^
Muscle relaxant	5/56	4/28	1/28	0.357^a^
Sedatives	6/56	3/28	3/28	1^a^

SD, standard deviation; BMI, body mass index; diagnostic, time since first diagnosis of fibromyalgia; TSK, tampa scale for kinesiophobia; DASH, disabilities of the arm, shoulder, and hand questionnaire; PCS, pain catastrophizing scale; WPI, widespread pain index; SS, symptom severity; FIQ, fibromyalgia impact questionnaire.

^a^ Based on Fisher's exact test.

^b^ Median (Q1-Q3.

^c^ Based on a Mann-Whitney *U*-test; antidepressants = duloxetine, sertraline, amitriptyline, venlafaxine, elavil, effexor, prozac, cymbalta, zoloft; anticonvulsants = pregabalin, gabapentin, epival, topiramate; analgesics = cymbalta, tramadol, tylenol, celecoxib, palmitoylethanolamide; muscle relaxants = cyclobenzaprine; sedatives = quetiapine, zopiclone.

### 3.2 Pain scores and PPT

#### 3.2.1 Changes in pain-related scores

**Figure 2:**
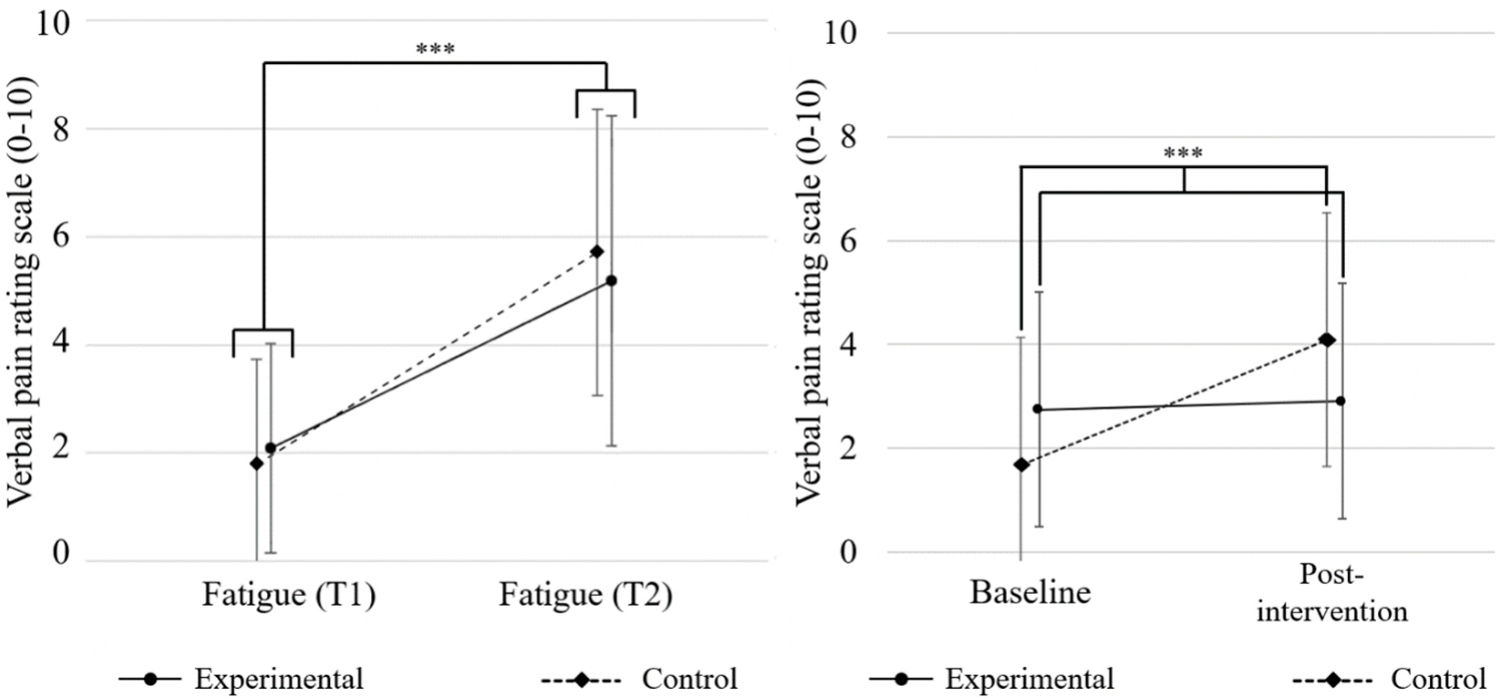
Comparison of fatigue (left) and visual rating pain scale of the middle deltoid (right) mean (SD). Error bars indicate standard deviation; **p ≤ 0.01.

Verbal pain rating scale and PPT scores are presented in Table 2. Results of the two-way repeated measures ANOVA for verbal pain rating scale indicated a significant Group*Time interaction effect for the middle deltoid muscle [F (1,54) = 12.846; *p* = 0.001; *η*_p_^2^ = 0.192] but not for the upper trapezius muscle [F (1,54) = 0.893; *p* = 0.349; *η*_p_^2^ = 0.016] and elbow extensor muscles [F (1,45) = 0.023; *p* = 0.88 *η*_p_^2^ = 0.001]. Significant changes in pain scores for the middle deltoid muscle are illustrated in Figure 2. Post-hoc Bonferroni test for multiple comparisons found that the mean value of verbal pain rating scale for the middle deltoid muscle of the control group increased significantly overtime compared to the experimental group following the repeated isometric muscle contraction task [*p *< 0.001, 95% CI = (−2.977, −1.380)]. A significant time effect was found for the upper trapezius [F (1,54) = 12.006; *p* = 0.001; *η*_p_^2^ = 0.182], middle deltoid [F (1,54) = 17.264; *p* < 0.001; *η*_p_^2^ = 0.242] and elbow extensors muscles [F (1,45) = 42.217; *p* < 0.001; *η*_p_^2^ = 0.484]. Verbal pain rating scores were higher following the repeated isometric muscle contraction task for the three areas. No significant Group effect was found for verbal pain rating scale values of the three evaluated muscles.

**Table 2:** Changes in verbal pain rating scale and PPT scores.

** **	Experimental (*n* = 28)		Control (*n* = 28)		*p*-value		
	Mean (± SD)		Mean (± SD)				
	Pre	Post	Pre	Post	G	T	G*T
VPRS (upper trapezius)/10	3.00 (±2.07)	3.71 (±2.72)	2.29 (±2.40)	3.54 (±2.55)	0.418	0.001***	0.349
VPRS (middle deltoid)/10	2.75 (±2.69)	2.91 (±2.83)	1.86 (±2.14)	4.04 (±2.38)	0.855	0.001***	0.001***
VPRS (elbow extensors)/10	3.58 (±2.55)	6.08 (±2.17)	2.87 (±2.26)	5.22(±2.13)	0.262	0.001***	0.88
PPT (upper trapezius)	2.75 (±1.28)	2.66 (±1.34)	2.46 (±0.90)	2.52 (±1.44)	0.434	0.988	0.460
PPT (middle deltoid)	3.25 (±1.85)	3.22 (±2.14)	3.01 (± 1.95)	2.96 (±1.95)	0.677	0.977	0.718
PPT (elbow extensors)	2.49 (±1.24)	2.67 (±1.87)	2.29 (±0.92)	2.52 (± 1.32)	0.553	0.100	0.953

SD, standard deviation; VPRS, verbal pain rating scale; PPT, pressure point threshold; Pre, baseline; Post, post-intervention; G, group effect; T, time effect; G*T, Group*Time interaction effect.

*** *p* ≤ 0.001.

Results of the two-way repeated measures ANOVA for PPT indicate no significant Time effect for the upper trapezius [F (1,51) = 0.011; *p* = 0.988; *η*p2 = 0], middle deltoid muscles [F (1,51) = 0.001; *p* = 0.977; *η*p2 = 0] and for elbow extensors [F (1,47) = 2.814; *p* = 0.100; *η*_p_^2^ = 0.056]. No significant Group or Group*Time effect was found for PPT scores of the three measured areas.

#### 3.2.2 Muscles fatigue during the repeated isometric muscle contraction task

The control group performed more repetitions (median: 19.5, 95% CI: 6; 30) than the experimental group (median: 16, 95% CI: 9; 30) during the fatigue task respectively), but this difference was not significant [t(52) = −0.885; *p* = 0.381]. Since participants did not perform the same number of total repetitions, fatigue scores at the beginning (T1) and at the end (T2) were compared. For fatigue, ANOVAs showed a significant effect of Time [F (1) = 130.01; *p *< 0.001; *η*_p_^2^ = 0.711] but no Group [F (1) = 0.002; *p *= 0.96; *η*_p_^2^ < 0.001] or Group×Time interaction effects [F (1) = 1.269; *p *= 0.27: *η*_p_^2^ = 0.023]. Both groups reported an increase in the fatigue score at the end of the fatigue task. Fatigue scores are presented in Figure 2.

### 3.3 EMG data

#### 3.3.1 Root mean square

The Mann-Whitney *U*-test showed no significant differences in RMS values during MVCs between the experimental group and the control group for the upper trapezius and the middle deltoid muscles (all *p* values >0.05). Median MVC RMS values of the upper trapezius muscle decreased from 0.13 mV (0.07–0.20) to 0.12 mV (0.07–0.16) for the experimental group and decreased from 0.14 mV (0.11–0.22) to 0.12 mV (0.10–0.19) for the control group. Mean MVC RMS values of the middle deltoid muscle decreased from 0.18 mV (0.11–0.31) to 0.15 mV (0.10–0.23) for the experimental group and decreased from 0.24 mV (0.16–0.30) to 0.15 mV (0.13–0.22) for the control group (see Table 3).

**Table 3:** Comparison of MVC RMS medians for upper trapezius and middle deltoid muscles between the two groups.

** **	** **	Experimental (*n* = 28)	Control (*n* = 28)	*p*-value	U value
RMS upper trapezius	Pre	0.13 (0.07–0.20)	0.14 (0.11–0.22)	0.381^a^	338
	Post	0.12 (0.07–0.16)	0.12 (0.10–0.19)	0.427^a^	343
RMS middle deltoid	Pre	0.18 (0.11–0.31)	0.24 (0.16–0.30)	0.225^a^	317.5
	Post	0.15 (0.10–0.23)	0.15 (0.13–0.22)	0.451^a^	347.5

Data are presented as median (Q1-Q3); RMS, root mean square.

^a^ Based on a Mann-Whitney *U*-test.

#### 3.3.2 Median frequency (MDF)

MDF values of the upper trapezius muscle were 84.63 (±9.69) and 84.74 (±10.69) at the beginning and end of the fatigue task for the experimental group and respectively 82.17 (±9.76) and 81.26 (±8.62) for the control group. The mixed model ANOVA showed that there was no significant difference in the MDF values between the experimental group and the control group for the upper trapezius muscle [F (1) = 1.36; *p* = 0.249; *η*_p_^2^ = 0.025]. No significant Time effect or Group*Time interaction effect was found for the upper trapezius muscle (all *p* values >0.05). MDF values of the middle deltoid muscle were 83.09 (±7.60) and 81.15 (±8.28) at the beginning and end of the fatigue task for the physical activity group and respectively 82.86 (±7.01) to 80.15 (±6.48) for the control group. No significant Group*Time interaction effect [F (1) = 0.319; *p* = 0.575; *η*_p_^2^ = 0.006] or Group was found [F (1) = 0.115; p = 0.736; *η*_p_^2^ = 0.002]. However a Time effect was observed for the middle deltoid muscle [F (1) = 10.948; *p* = 0.001; *η*_p_^2^ = 0.174]. MDF scores are presented in Table 4.

**Table 4:** Comparison of the first and last median frequency for the upper trapezius and middle deltoid.

** **	Experimental (*n* = 26)	Control (*n* = 28)	*p*-value		
			Group	Time	G*T
First MDF upper trapezius	84.63 (±9.69)	82.17 (±9.76)	0.249	0.573	0.476
Last MDF upper trapezius	84.74 (±10.69)	81.26 (±8.62)			
First MDF middle deltoid	83.09 (±7.60)	82.86 (±7.01)	0.736	0.001**	0.576
Last MDF middle deltoid	81.15 (±8.28)	80.15 (±6.48)			

Data are presented as mean (± SD); MDF, median frequency.

** *p* ≤ 0.01.

### 3.4 Force data

**Figure 3:**
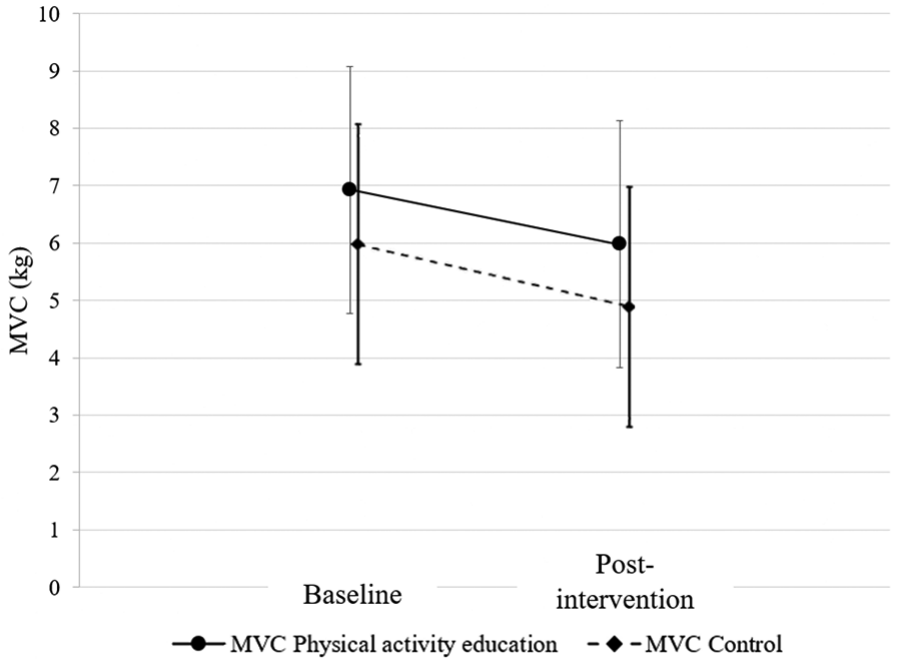
Comparison of baseline and post-intervention average force during maximal contraction between the physical activity education and control groups. MVC, maximal voluntary contraction.

On average, the experimental group showed higher strength values than the control group, both for baseline (6.93 kg (±2.38) vs. 5.98 kg (±2.02)) and post-intervention (5.98 kg (±2.01) vs. 4.92 kg (±2.08)) values. Mixed model ANOVAs showed no significant effect of Group [F (1) = 3.391; *p* = 0.071; *η*_p_^2^ = 0.059] and Group*Time [F (1) = 0.114; *p* = 0.737; *η*_p_^2^ = 0.002]. Only a Time effect was found, as both groups showed a non-significantly different decrease in force of −0.95 (±1.58) kg for experimental group and −1.06 (±0.94) kg for control group [F (1) = 33.396; *p* < 0.001; *η*_p_^2^ = 0.382]. Force data are presented in Figure 3.

## 4 Discussion

The objective of this pilot study was to assess how a 5-minute session of physical activity education impacts the development of shoulder pain in specific areas (upper trapezius, middle deltoid, and elbow extensor muscles) following repeated upper limb contractions among individuals with fibromyalgia. We hypothesized that pain and fatigue would be positively impacted by a 5-minute physical activity education session. Furthermore, we expected a smaller MVC reduction following the physical activity education session for the intervention group.

As expected, our preliminary results for the verbal pain rating scale values were significantly influenced by the physical activity education session but only for the middle deltoid area. Verbal pain rating scale scores increased for the control group following the intervention, but the 5-minute physical activity education session appeared to limit the increase in pain for the intervention group. On the other hand, PPTs increased similarly in both groups as analyses showed no significant between-group differences in PPTs scores for all tested areas, indicating that the intervention did not influence PPT for the experimental group. The change in the fatigue scores between the beginning and the end of the fatigue task were also not significantly different between groups.

Interestingly, the EMG analyses showed that, although a slight decrease in the MDF values of the two evaluated muscles was observed following the fatigue tasks, this effect was not significant suggesting that no muscle fatigue was developed during the task. No significant difference between the experimental group and the control group was found for MVC values following the intervention. However, both groups showed a significant decrease in force after the intervention.

Our preliminary results are in accordance with recent studies showing a positive impact of education on pain (27, 44, 45). Especially, the recent meta-analysis of Suso-Marti et al. (27) highlighted a significant positive effect of education with a moderate clinical effect on pain intensity at post-intervention. However, these studies focused on education programs exploring various themes (neuroscience of pain and good lifestyle habits) and did not specifically focus on physical activity and kinesiophobia as addressed in our study. Comparing the impact of our short-term education data to that of long-term protocols is complex, making it challenging to draw direct parallels between their respective outcomes. But it appears that interventions with either a general or a targeted physical activity theme result in similar effects on pain, despite a shorter intervention duration. The duration of the education programs in recent scientific evidence ranged from 4 to 12 weeks (27). It is therefore necessary to study the effect of multiple physical activity education sessions with a long-term follow-up.

In this pilot study, physical activity education influenced verbal pain rating scale scores, but not strength or muscular fatigue. Consistent with our results, Rooks et al. (46) reported that upper limb strength of participants with fibromyalgia did not significantly change after a 7 session health education program. A systematic review by Garcia-Rios et al. (47) found that the most common findings in studies investigating the effect of health education are improved patient beliefs about pain management. Participants in both of our groups did present clinically significant baseline levels of kinesiophobia (≥37/68) (48), which may suggest that patients’ pain management beliefs, rather than physical or physiological changes, may account for the reduction in pain scores following a single physical activity education session. Overall, it is recommended to integrate health education with exercise to optimize the benefits of patient management for symptoms associated with fibromyalgia (46, 47). As suggested by Araujo and DeSantana (49), the treatment of fibromyalgia should include both pharmacological and non-pharmacological interventions. Active physical treatments such as aerobic exercise, muscle strengthening, stretching, and aquatic therapy have demonstrated beneficial effects on fibromyalgia symptoms including improved quality of life, pain and fatigue reduction (49). Pharmacological treatments for pain management typically include pain relievers, antidepressants, and/or anticonvulsants (15). Additionally, interventions such as vitamin D supplementation (50) or mesotherapy (51), can be used to decrease pain and enhance the quality of life of patients with fibromyalgia.

The PPT analysis showed no significant changes in scores before and after repeated upper limb contractions. Our results are consistent with those of Falla et al. (40) who found no significant augmentation of PPT scores after a sustained isometric contraction in fibromyalgia participants. Moreover, Hoeger Bement et al. (52) investigated the effectiveness of isometric exercise protocols (muscular contraction similar to those used in our pilot study) on short-term hypoalgesia in patients with fibromyalgia. Due to the heterogeneity of the results across patients, the authors found no significant change in pain measured by PPT. The variability in pain responses following isometric exercise has been found in prior research (53). The isometric contraction needs to be intense to trigger adaptation mechanisms in pain for fibromyalgia participants (17). It is likely that the isometric contraction in our pilot study may not have been sustained or intense enough to trigger central sensitization.

Generating upper limb MVC can be challenging for patients with fibromyalgia as it is for most patients with chronic pain conditions (23). Regarding physical and physiological outcomes, it is well known that muscle strength can be influenced by pain, fear of pain (23) or kinesiophobia (54), and that these psychological factors can lead to lower MVC in fibromyalgia patients compared to healthy participants (23). Although a significant decrease in MVC was observed in the current pilot study following repeated upper-limb contractions, EMG analyses showed that neither participants in the intervention group nor the control group experienced muscle fatigue following the fatigue task. Bandak et al. (55) showed that patients with fibromyalgia perceive more muscle fatigue at lower objective muscle fatigue levels (determined by muscle MDF and RMS) than healthy participants, suggesting that there might be a partial disconnect between perceived muscle fatigue and physiological biomarkers of muscle fatigue, as shown by our results. Furthermore, the difference between perceived and objective muscle fatigue may prevent fibromyalgia patients from obtaining beneficial muscle stimuli from exercise.

Our analyses demonstrate that the measurement of fatigue and muscle pain in participants with fibromyalgia is relevant, and that a larger-scale study would allow for a more precise estimate of the treatment effect. As this is a pilot study, our results and available scientific evidence suggest that a potential full-scale trial should include confounding factors such as psychological variables.

## 5 Clinical application

The preliminary results of this study suggest that education to physical activity and kinesiophobia, could potentially be integrated into the management of patients with fibromyalgia. Sharing knowledge about the pathology and its cardinal signs and symptoms to patients with fibromyalgia could also increase self-efficacy and self-management strategies while improving overall lifestyle, reducing fear and dramatization of pain (1, 47). A 5-minute session is certainly not sufficient to improve pain and quality of life in these patients on a lasting basis. Larger clinical trials investigating the efficacy of physical activity education are therefore warranted. Such trials could be designed according to current evidence showing that the effects of cognitive-behavioral therapy (the gold standard of psychological treatment) on core fibromyalgia symptoms are significant after 5–25 h of therapy and increase after more than 75 h of treatment (1, 56). A meta-analysis by Suso-Marti et al. (27) showed that the total duration of pain neuroscience education ranges from 20 min (57) to 16 h (58). Future studies should focus on increasing the duration of fibromyalgia educational strategies including physical activity education, combined with other interventions. The ongoing challenges of fibromyalgia as a public health issue requires an increased focus on health education in the treatment of the pathology. This could involve empowering individuals with knowledge on health promotion, symptom self-management, and appropriate treatment. Like the example of *Fibro Friends* in Brazil (30), this health education program aim to equip individuals with fibromyalgia with the skills to recognize pain triggers, navigate illness challenges, and cultivate autonomy through self-discovery and lifestyle adjustments for health maintenance and improvement. The outcomes of these programs could potentially reduce the overall burden on the healthcare system and promoting cost-effective, patient-centered care.

### 5.1 Limitations identified on the pilot study

The sample of participants recruited in our study may not be representative of the average worldwide fibromyalgia population as the average worldwide female-to-male ratio of fibromyalgia is 3:1 (1) but women represented 98.3% of the study sample. Although such bias in recruitment cannot be explained by our results, it has also been observed in other studies (59). Other researchers have suggested that fibromyalgia is more prevalent in women because of inherent bias in criteria related to symptom severity, referral, and diagnostic criteria (60).

Moreover, our pilot study was conducted in a laboratory setting and investigated only short effect of physical activity education. Therefore, caution should be exercised when generalizing the results to activities of daily living and physical activity of patients living with fibromyalgia. Subsequent studies with larger samples and longer observation periods are needed to confirm our findings.

## 6 Conclusion

The results of this pilot study showed that a 5-minute physical activity education video positively influenced middle deltoid pain of participants with fibromyalgia following shoulder abductions. These preliminary findings emphasize the significant potential of educational interventions in managing fibromyalgia, offering promising prospects for enhancing the quality of life of individuals affected by this condition. Furthermore, it is important to highlight the role of health education as a fundamental tool for promoting health in individuals with fibromyalgia. It is essential to recommend long-term programs aimed at reducing the impact of fibromyalgia symptoms, particularly concerning pain and muscle fatigue.

Therefore, it would be relevant to study the effects of physical activity education on long-term management of upper limb pain and dysfunction on participant with fibromyalgia. It also seems necessary to explore inconsistencies between perceived muscle fatigue and physiological markers of muscle fatigue in fibromyalgia participants.

## Data Availability

The raw data supporting the conclusions of this article will be made available by the authors, without undue reservation.
